# Timing of Newborn Hearing Screening Effects on Passing Rates: A Prospective Cohort Study

**DOI:** 10.1055/a-2675-1768

**Published:** 2025-08-20

**Authors:** Wongsathon Seehiranwong, Pichada Saengrat

**Affiliations:** 1 Department of Pediatrics, Faculty of Medicine, Vajira Hospital, Navamindradhiraj University, Bangkok, Thailand

**Keywords:** newborn hearing screening, otoacoustic emission, screening timing, TEOAE

## Abstract

**Objectives:**

Newborn hearing screening using transient evoked otoacoustic emissions (TEOAEs) is essential for early detection of hearing impairment. The Joint Committee on Infant Hearing recommends screening near hospital discharge but does not specify an optimal timing. To determine the optimal timing for TEOAE screening in neonates at low risk of hearing impairment and to identify perinatal factors influencing pass rates.

**Study Design:**

Neonates underwent sequential TEOAE screening based on postnatal age at the time of testing: less than 24, 24 to 36, 36 to 48, and more than 48 hours, with follow-up at 1 month for persistent failures. Statistical analyses included Fisher's exact test to compare pass rates across time intervals and multivariate Cox's proportional hazards regression and Laplace regression to assess factors associated with screening outcomes.

**Results:**

Among 408 neonates, the median passing age was 23.8 hours (interquartile range: 14.3). Pass rates improved with later screening: 53.7% at less than 24 hours, 80.1% at 24 to 36 hours, 92.6% at 36 to 48 hours, and 99.3% at more than 48 hours. A significant improvement was observed only at more than 48 hours (odds ratio: 5.26; *p* = 0.0153). Cesarean delivery was associated with delayed passing compared with vaginal delivery ( *p* = 0.036). Late preterm neonates demonstrated a significantly delayed passing time of approximately 12.9 hours ( *p* < 0.01), whereas small for gestational age neonates passed earlier by 8.2 hours ( *p* = 0.021).

**Conclusion:**

Screening at greater than or equal to 48 hours significantly improved pass rates. An older age at successful screening was observed among preterm neonates and those delivered by cesarean section, underscoring the need for tailored follow-up protocols. These findings highlight the importance of optimizing screening strategies to enhance early detection and intervention.

**Key Points:**

- The ideal timing for newborn hearing screening remains unclear, affecting accuracy and follow-up rates.
- Early TEOAE screening may lead to high false positives due to residual ear fluid, increasing unnecessary follow-ups.
- Optimizing timing of the hearing screening window can enhance accuracy, reduce false positives, and improve clinical efficiency.

Hearing is vital for communication and the development of speech, language, and social skills. 1 2 Early detection and intervention within 6 months are essential for preventing developmental delays. 2 Hearing loss affects 430 million people globally (5% of the population), with 0.5 to 5 cases per 1,000 live births worldwide and in Thailand, underscoring the importance of neonatal hearing screening (NHS). 3 4 5 6 7 8 A Greek study 7 highlighted the benefits of universal NHS in countries like the United States and Australia, where it has improved early diagnosis, language development, and education. However, implementation across Europe remains inconsistent. Thailand still faces challenges in achieving nationwide NHS coverage.

Screening methods include otoacoustic emissions (OAEs), automated auditory brainstem response (AABR), two-tier protocols (OAEs followed by AABR), and combined OAEs and AABR. OAEs are cost-effective but prone to higher referral rates, while AABR is more accurate but expensive. ABR sensitivity ranges from 45 to 100%, specificity 71.3 to 99.3%, with referral rates less than 2%. 9 OAEs show sensitivity of 96 to 100%, specificity of 91%, and referral rates of 6.5 to 13%. 8 Vajira Hospital is one of the hospitals under the Bangkok metropolitan administration network, which includes several tertiary and secondary care hospitals. This network aims to provide healthcare services to the population of Bangkok and its surrounding metropolitan area. Our center, a university-affiliated tertiary care hospital, along with most hospitals in Thailand, uses OAEs as the primary newborn hearing screening method because it is more affordable and easier to perform than AABR.

OAEs detect sound emissions from outer hair cells using an ear probe. However, residual amniotic fluid or middle-ear fluid can cause false positives, increasing referrals. OAEs also miss auditory neuropathy and central pathway abnormalities. 8 9 10 11

In October 2022, 32% of 114 newborns with transient evoked otoacoustic emission (TEOAE) screened in our hospital's neonatal ward were referred for repeat testing. Most screenings (72%) occurred within 24 hours of birth, contributing to the high referral rate, consistent with global findings.

False-positive results heighten parental anxiety, potentially leading to vulnerable child syndrome, affecting child development and family dynamics. Repeat testing adds financial and emotional strain to families and healthcare systems. 12 Early screenings (<24 hours) have higher false-positive rates. For example, Lupoli et al 13 found false positives at 42.4% within 24 hours versus 24.68% after 24 hours ( *p* < 0.001), with rates dropping 5% per hour. Xiao et al 14 reported false-positive rates of 45.4% before 24 hours and 3.1% after 48 hours. Cesarean deliveries also show higher failure rates, normalizing after 42 hours. 14 15

Some centers used ABR screening as a primary method of screening. A recent study, Oruç et al 16 studied the NHS in Turkey, screening 5,399 infants using ABR before discharge. Ninety-six point nine percent passed the first step, and 2.5% required further testing. Bennett et al 17 found ABR screening led to earlier auditory neuropathy diagnosis (14.1 weeks vs. 27.3 weeks; *p* = 0.0397) than OAE, supporting universal ABR use for timely intervention, especially in high-risk infants.

Dimitriou et al 18 conducted a prospective cohort study in Greece and found the highest pass rates for the NHS on days 3 (90%) and 4 (94%). In Asia, a Korean retrospective cohort study 19 found the lowest referral rates in well babies screened between 2 and 20 days, while later screenings (>60 days) increased referrals without affecting hearing loss incidence. A systematic review in China revealed that the timing of the first NHS varies across countries. Most recommend screening before discharge—within the first few days or weeks of life using OAE or AABR—with rescreening typically occurring within 1 to 3 months, except in India (4–6 weeks during immunization visits) and England (4–5 weeks). 20 The Joint Committee on Infant Hearing (JCIH) recommends screenings near hospital discharge with time for repeats, but provides no specific timing. 8 In Thailand, including at our center, most healthy term newborns are discharged together with their mothers approximately 48 hours after vaginal delivery and 72 hours after cesarean section, provided there are no complications. Despite evidence supporting delayed screening to reduce false positives, Southeast Asian research remains limited.

This prospective study uses survival analysis to examine the timing of newborn hearing screenings and pass rates in Thailand, offering new insights to optimize protocols, reduce false positives, and improve outcomes for families and healthcare systems.

## Materials and Methods

### Study Design and Participants

This prospective cohort study evaluated the optimal timing for TEOAE screening in newborns admitted to the well neonatal ward at our center, which is the tertiary care center, from September 2023 to April 2024. Newborns with parental consent were included, excluding those transferred to intensive care or classified as high-risk based on JCIH 2019 criteria (family history of childhood hearing loss, prolonged NICU stay, hyperbilirubinemia requiring exchange transfusion, ototoxic drug use, hypoxic-ischemic encephalopathy, ECMO therapy, intrauterine infections, congenital anomalies, syndromic conditions associated with hearing loss, postnatal infections, and significant events such as head trauma or chemotherapy exposure). 8 21

The sample size was calculated using Fleiss' method, 22 requiring 366 participants to detect differences between screening groups, adjusted to 400 to account for an expected 10% dropout rate. Ethical approval was granted by the institutional review board, Faculty of Medicine (COA 063/2566).

The screening tool used was the Madsen AccuScreen TEOAEs device, which detects inner ear responses to sound stimuli for the NHS. The study strictly adhered to the manufacturer's criteria, requiring artifacts to be less than 20% and stimulus stability more than 80% for valid results. Pass or refer decisions were based on a stimulus level more than 75 ± 5 dB with more than or equal to eight valid peaks per ear. If these criteria were not met, the device provided troubleshooting suggestions, such as waiting for quieter conditions or adjusting probe fit. 23

Eligible newborns were sequentially screened by a trained assistant nurse in low-noise conditions from birth until discharge at five intervals: within 24, 24 to 36, 36 to 48, beyond 48 hours, and at 1 month for persistent failures (conducted by an audiologist at the otolaryngology outpatient department). If newborns passed the NHS at an earlier interval, they were assumed to have passed all subsequent intervals due to the high sensitivity of the TEOAEs tool. Persistent failures were referred for audiological evaluation, including tympanometry and AABR. Diagnosed cases were referred for appropriate management.

### Statistical Analysis

Data on screening outcomes, demographics, and testing intervals were recorded in an encrypted database with anonymized identifiers. Statistical analysis was performed using STATA 18.0 (Stata Corp, United States). Continuous variables were summarized as mean ± standard deviation (SD) or median (interquartile range, IQR), and categorical variables as frequencies, percentage (%). Referral rates across four screening age groups (<24, 24–36, 36–48, and >48 hours) were compared using Fisher's exact test ( *p* < 0.05).

Survival analysis, including Cox and Laplace regression 24 25 was used to assess the age at passing TEOAEs and covariates such as delivery method, gender, gestational age (GA), birth weight (BW), and other perinatal risk factors. Outcomes included hazard ratios with 95% confidence intervals, survival time percentiles (25th, 50th, 75th, and 90th), and differences in survival age for each covariate across these percentiles.

## Results

### Demographic Data

The study included a total of 408 eligible subjects with complete follow-up. The median passing age for hearing screening was 23.8 hours (IQR: 14.3). Most infants (95.1%) had an APGAR score of 10 at 5 minutes, indicating no perinatal asphyxia. The majority were born at GA greater than or equal to 37 weeks (81.9%) with a median GA of 38.7 weeks (IQR: 1.3) and BW of 3,030 g (IQR: 526.2). A small proportion had low birth weight (LBW; 6.4% were <2,500 g), with 8.3% classified as small for gestational age (SGA), 4.2% as large for gestational age (LGA), and 87.5% as appropriate for gestational age (AGA). In terms of delivery mode, 34.1% were delivered via cesarean delivery, while 65.9% were delivered via vaginal delivery. Persistent failure requiring rescreening was found in three cases (0.7%). All three infants subsequently passed both TEOAEs and AABR screening at the age of 1 month. Following successful rescreening, none of the infants have been diagnosed with permanent hearing loss to date. Noise and artifact levels were low (mean: 0.9%; SD: 1.9), with all values below 20%. Stability was high (mean: 94%; SD: 2.22), with all values exceeding 80%. These findings indicate effective environmental control, as the screening tool defines satisfactory quality as artifact less than 20% and stability more than 80%. Detailed demographic data and results are provided in Table 1 .

**Table 1:** Demographic data and univariate Cox's regression analysis

Total eligible subjects, *n*		408	Gestational age (wk), median (IQR)	38.7 (1.3)
Passing age (h), median (IQR)		23.8 (14.3)	Birth weight (g), Median (IQR)	3,030 (526.2)
Male, *n* (%)		218 (53.43%)	Final passing rate	408 (100%)
** ** Category	*n* (%)	Median passing age (IQR; h)	HR (95% CI) a	*p* -Value b
APGAR score at 5 min				
8–9	23 (5.64)	33.2 (20.8)	0.74 (0.48–1.13)	0.172
10	388 (95.1)	23.7 (13.9)	Reference	–
Gestational age (wk)				
35–37	20 (4.9)	24.7 (17.1)	0.68 (0.42–1.10)	0.119
≥37	334 (81.86)	23.7 (14.5)	Reference	–
Birth weight (g)				
2,200–2,500	26 (6.37)	25.6 (22.6)	0.70 (0.46–1.07)	0.09
≥2,500	382 (93.63)	23.7 (14.3)	Reference	–
Birth weight for gestational age				
SGA	34 (8.33)	23.8 (14.9)	1.17 (0.82–1.67)	0.377
LGA	17 (4.17)	23.5 (10.2)	1.01 (0.62–1.65)	0.969
AGA	357 (87.5)	23.2 (16.2)	Reference	–
Mode of delivery				
Cesarean delivery	139 (34.07)	24.0 (11.1)	0.79 (0.64–0.99)	0.036
Vaginal delivery	269 (65.93)	23.5 (15.8)	Reference	–
Other perinatal risk factors				
No other risk factor	172 (42.16)	22.8 (14.5)	Reference	–
Maternal risk factors				
Maternal gestational diabetes mellitus	57 (13.97)	24.8 (15.3)	0.89 (0.66–1.19)	0.432
Advanced maternal age	40 (9.8)	25.0 (20.0)	0.72 (0.518–1.01)	0.057
Teenage mother	24 (5.88)	25.4 (14.9)	1.05 (0.69–1.59)	0.822
Maternal obesity	15 (3.68)	30.6 (18.2)	0.98 (0.58–1.64)	0.939
Maternal syphilis (adequate treatment)	2 (0.49)	45.2 (32.6)	NA	–
Maternal drug use	1 (0.25)	14.5 (−)	NA	–
Maternal hyperthyroid	1 (0.25)	33.4 (−)	NA	–
Placental risk factors				
Thick MSAF	30 (7.35)	23.8 (14.1)	0.98 (0.67–1.44)	0.917
Placenta previa	4 (0.98)	24.4 (14.9)	NA	–
Oligo- or Anhydramnios	4 (0.98)	38.4 (18.2)	NA	–
Polyhydramnios	1 (0.25)	22.3 (−)	NA	–
Infant risk factors				
Neonatal jaundice	95 (23.28)	23.6 (17.7)	0.86 (0.67–1.09)	0.216
Respiratory distress	64 (15.69)	25.9 (28.6)	0.95 (0.71–1.26)	0.702
Twins	2 (0.49)	48.0 (6.4)	NA	–
Perinatal event				
FHR nonreassuring c	7 (1.72)	35.1 (14.1)	0.72 (0.33–1.59)	0.417
Birth before arrival	3 (0.74)	21.4 (18.1)	NA	–

Abbreviations: AGA, appropriate for gestational age; FHR, fetal heart rate; LGA, large for gestational age; MSAF, meconium-stained amniotic fluid; NA, not applicable; SGA, small for gestational age.

Note: NA means not applicable due to an insufficient number of cases for statistical analysis.

a Hazard ratios with 95% confidence intervals are derived from univariate Cox's regression analysis.

b *p* -Value is obtained from the Cox's regression analysis with statistically significant at *p* < 0.05.

c FHR nonreassuring includes abnormal fetal heart rate patterns indicative of possible fetal distress.

### Comparison of Screening Intervals

**Fig. 1:**
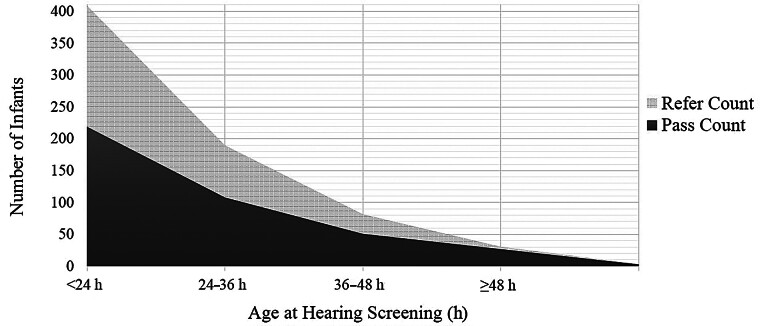
A stacked area graph displaying the cumulative number of infants who passed the hearing screening (darker area) across different age groups: <24, 24 to 36, 36 to 48, and ≥48 hours. The accumulated pass rate increased progressively with screening age, starting at 53.7% for those screened before 24 hours, rising to 80.1% at 24 to 36 hours, 92.6% at 36 to 48 hours, and reaching 99.3% in the ≥48-hour group.

The pass rate improved as the screening time increased ( Table 2 ). Among newborns screened before 24 hours of age, 53.7% passed, while 46.3% required further testing. Screening between 24 and 36 hours resulted in a slightly higher pass rate of 57.1% (odds ratio [OR]: 1.15; 95% CI: 0.83–1.63; *p* = 1.00) compared with those screened before 24 hours. At 36 to 48 hours, the pass rate further increased to 62.9% (OR: 1.28; 95% CI: 0.75–2.18; *p* = 1.0000). Newborns screened at or beyond 48 hours demonstrated a significantly higher pass rate of 90.0% compared with those screened at 36 to 48 hours (OR: 5.26; 95% CI: 1.48–18.95; *p* = 0.0153). The accumulated pass rate increased progressively with screening age, as shown in Fig. 1 . This trend highlights a marked improvement in pass rates with delayed screening. A total of three newborns required re-screening, all of whom subsequently passed. These findings suggest that delaying initial NHS beyond the first 48 hours may reduce false-positive referrals.

**Table 2:** TEOAEs pass rates by screening age group (Fisher's exact test)

Age groups (h)	Total	Pass (%)	Refer (%)	Accumulated pass (%)	Comparisons to (h)	OR (95% CI) a	*p* -Value b
<24	408	219 (53.7)	189 (46.3)	219 (53.7)	–	–	–
24–36	189	108 (57.1)	81 (42.8)	327 (80.1)	<24	1.15 (0.83–1.63)	1
36–48	81	51 (62.9)	30 (37.0)	378 (92.6)	24–36	1.28 (0.75–2.18)	1
≥48	30	27 (90.0)	3 (10.0)	405 (99.3)	36–48	5.26 (1.48–18.95)	0.0153
Need for re-screening	3	3 (100.0)	0 (0.0)	408 (100)	NA	NA	NA

Abbreviations: CI, confidence interval; OR, odds ratio; TEOAEs, transient evoked otoacoustic emissions.

a Statistically significant at *p* < 0.05.

a Odds ratios with 95% confidence interval from Fisher's exact test.

b Bonferroni-adjusted *p* -values (α = 3; significance: *p* < 0.05).

### Analysis of Perinatal Factors Using Cox's Proportional Regression Analysis

Cox's proportional regression analysis assessed associations between maternal, neonatal, and perinatal factors with TEOAEs passage times. Factors excluded due to small sample sizes were maternal syphilis (adequate treatment), maternal drug use, maternal hyperthyroidism, placenta previa, oligohydramnios or anhydramnios, polyhydramnios, twins, and birth before arrival. Relevant factors underwent univariate analysis, followed by multivariate analysis using the backward stepwise method, selecting the optimal model based Akaike information criterion and Bayesian information criterion. The final model included LBW, preterm birth, cesarean delivery, and neonatal jaundice. The proportional-hazards assumption was met ( *p* = 0.263), confirming model validity.

**Fig. 2:**
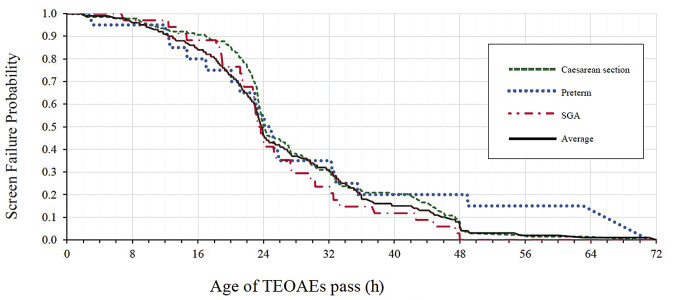
Show Cox's regression curves for the passing times of TEOAE screening by mode of delivery, birth weight for GA, and preterm newborns. Cesarean delivery showed a delay in passing within the first 24 hours ( *p* = 0.024), but the rates converged afterward. SGA newborns have a better passing rate than appropriate for AGA newborns within 48 hours, and preterm newborns experience a significant delay in passing after the 24-hour period. AGA, appropriate for gestational age; SGA, small for gestational age; TEOAEs, Transient evoked otoacoustic emissions.

Cesarean delivery was associated with a mild but significant delay in TEOAE passage compared with vaginal delivery (median: 24.0 hours vs. 23.5 hours), with hazards ratio (HR): 0.79 (95% CI: 0.64–0.99; *p* = 0.036) in univariate analysis and HR: 0.77 (95% CI: 0.62–0.96; *p* = 0.02) in multivariate analysis. Neonates with an APGAR score of 10 at 5 minutes passed TEOAEs at a median age of 23.7 hours, slightly earlier than those with scores of 8 to 9 (33.2 hours), though this factor was not significantly associated with TEOAE outcomes. GA and BW were not significantly associated with TEOAE passage times (HR: 0.68; 95% CI: 0.42–1.10; *p* = 0.119; and HR: 0.70; 95% CI: 0.46–1.07; *p* = 0.09, respectively). Similarly, BW for GA categories (SGA, AGA, and LGA) showed no notable differences ( *p* > 0.4). Neonates with perinatal risk factors passed TEOAEs slightly later than those without (median: 24.3 hours vs. 22.8 hours), but specific risks such as respiratory distress and neonatal jaundice were not significantly associated with TEOAE outcomes ( *p* > 0.05). Other maternal and neonatal factors, including advanced maternal age, obesity, and obstetric complications, showed no significant trends, though small sample sizes limited detailed analysis. Further details are presented in Tables 1 and 3 and Figs. 1 and 2 .

**Table 3:** Multivariate Cox's regression analysis for time to TEOAE pass

Variable	Hazard ratio	95% CI	*p* -Value a
Low birth weight	0.76	0.48–1.19	0.232
Neonatal jaundice	0.85	0.67–1.08	0.174
Cesarean delivery	0.77	0.62–0.96	0.020
Preterm	0.75	0.44–1.26	0.271

Abbreviations: CI, confidence interval; TEOAE, transient evoked otoacoustic emission.

a Statistically significant at *p* < 0.05.

### Comparing between First Hearing Screening Interval

When comparing hearing screening outcomes across age groups, significant differences were observed in neonates screened at more than 48 hours. Neonates screened at more than 48 hours had substantially higher odds of passing TEOAEs compared with those screened at earlier time points (0–23.99 hours; OR: 7.77; 95% CI: 2.32–26.01; adjusted *p* < 0.01; 24–35.99 hours; OR: 6.75; 95% CI: 1.98–23.03; adjusted *p* < 0.01; and 36–47.99 hours: OR: 5.29; 95% CI: 1.48–18.95; adjusted *p* < 0.01). No significant differences were found between the earlier age groups (0–23.99 vs. 24–35.99; OR: 1.15; adjusted *p* = 1.00; 0–23.99 vs. 36–47.99; OR: 1.47; adjusted *p* = 0.855; and 24–35.99 vs. 36–47.99; OR: 1.28; adjusted *p* = 1.00). Detailed comparisons are presented in Table 2 .

### Analysis of Procedural Factors

Hearing screening outcomes varied slightly by location and investigator. Neonates screened in Screening room-1 had a significantly higher likelihood of passing TEOAEs (HR: 1.54; 95% CI: 1.25–1.90; *p* < 0.01) compared with other rooms, while rooms 2 and 3 showed no significant differences ( *p* = 0.568 and *p* = 0.463, respectively). No significant associations were identified for outcomes across individual investigators (all *p* > 0.3), with hazard ratios ranging from 0.90 to 1.44.

### Laplace Regression

Multivariate analysis using Laplace regression revealed the following significant findings: Preterm neonates exhibited a significantly delayed 90th percentile age for passing TEOAEs compared with term neonates, with a difference of 12.9 hours (95% CI: 5.1–20.7; *p* < 0.01). Neonates delivered via cesarean section showed older ages at the 25th and 50th percentiles (6.1 hours; 95% CI: 2.8–9.4; *p* < 0.01; 3.1 hours; 95% CI: 0.6–5.7; *p* = 0.017, respectively) compared with those born vaginally. SGA neonates had a significantly earlier 90th percentile age (−8.2 hours; 95% CI: −15.1 to −1.2; *p* = 0.021). No significant differences in hearing screening timing were observed across other variables, including neonatal jaundice, LBW, or respiratory distress (all *p* > 0.05). Detailed data are provided in Table 4 .

**Table 4:** Multivariate analysis using Laplace regression in the quantiles of 25th, 50th, 75th, and 90th percentile a

	25th percentile			50th percentile			75th percentile			90th percentile		
Factors	Mean age	95% CI	*p* -Value	Mean age	95% CI	*p* -Value	Mean age	95% CI	*p* -Value	Mean age	95% CI	*p* -Value
Reference	22.3	NA	NA	28.8	(24.7–32.9)	NA	32.9	(22.3–43.5)	NA	45.4	(32.6–58.2)	NA
Male	22.5	(−2.0 to 2.5)	0.810	29.8	(−1.8 to 3.7)	0.500	35.1	(−4.6, 9.0)	0.525	44.8	(−6.4, 5.1)	0.827
Preterm	23.6	(−9.9 to 12.5)	0.816	28.3	(−13.5 to 12.4)	0.936	33.5	(−25.5, 26.8)	0.961	58.3	(5.1, 20.7)	<0.01
LBW	25.8	(−1.4 to 8.5)	0.163	32.0	(−4.8 to 11.2)	0.431	40.9	(−8.5, 24.6)	0.342	53.9	(−6.0, 22.9)	0.250
SGA	22.7	(−3.8 to 4.6)	0.844	28.4	(−6.5 to 5.8)	0.905	31.9	(−8.1, 6.1)	0.779	37.2	(−15.1 to 1.2)	0.021
LGA	23.5	(−3.9 to 6.5)	0.632	27.6	(−4.4 to 2.1)	0.474	35.3	(−30.7, 35.5)	0.888	48.5	(−27.5, 33.7)	0.844
Cesarean delivery	28.4	(2.8–9.4)	<0.01	31.9	(0.6–5.7)	0.017	35.7	(−2.6, 8.2)	0.312	49.9	(−9.9, 19.0)	0.540
IDM	22.4	(−3.0 to 3.3)	0.927	29.6	(−2.6 to 4.3)	0.636	39.9	(−3.2, 17.4)	0.179	44.1	(−9.2, 6.5)	0.739
Thick MSAF	24.2	(−3.3 to 7.2)	0.460	29.5	(−3.3 to 4.8)	0.721	31.9	(−11.0, 9.1)	0.853	46.5	(−8.4, 10.5)	0.825
FHR nonreassuring	29.6	(−0.5 to 15.2)	0.066	34.7	(−7.4 to 19.2)	0.388	41.0	(−10.8, 27.1)	0.399	51.8	(−25.8, 38.5)	0.698
Neonatal jaundice	21.0	(−6.1 to 3.5)	0.604	30.9	(−1.9 to 6.1)	0.312	35.5	(−9.9, 15.2)	0.683	49.6	(−10.1, 18.5)	0.565
Respiratory distress	24.3	(−1.0 to 5.0)	0.191	30.5	(−0.9 to 4.2)	0.196	34.2	(−6.7, 9.3)	0.747	41.1	(−13.7, 5.0)	0.366

Abbreviations: CI. Confidence interval; FHR, fetal heart rate; IDM, infants of diabetic mothers; LBW, low birth weight; LGA, large for gestational age; MSAF, meconium-stained amniotic fluid; SGA, small for gestational age.

a Multivariate Laplace regression analysis was performed using key variables including patient demographics (sex, gestational age, birthweight, and birthweight for gestational age), mode of delivery, perinatal risk factors (IDM, respiratory distress, nonreassuring fetal heart rate, and neonatal jaundice), and procedural factors (investigator and screening room).

## Discussion

### Optimizing the Timing of TEOAE Screening

This study provides insights into optimizing TEOAEs screening timing and highlights key perinatal and demographic factors influencing outcomes. Screening after 48 hours showed significantly higher passing rates (>99%) compared with earlier screenings (OR: 5.26; 95% CI: 1.48–18.95; *p* < 0.05), suggesting that delayed screening is a more reliable approach. A 36 to 48-hour interval (80–92% passing rate) appears to offer a reasonable balance between clinically practical and maintaining reliability, with rescreening at 48 to 72 hours as an option for confirmation. Screening before 36 hours, with a passing rate less than 80%, may not be as effective due to lower success rates.

These findings align with previous studies by Lupoli et al, 13 Xiao et al, 14 and Dimitriou et al, 18 which similarly reported reduced false positives with delayed screening. The cohort's median passing age of 23.7 hours reflects current institutional protocols and demographic characteristics. Additionally, this study suggests that personalized screening, particularly for preterm and cesarean-delivered neonates, could improve accuracy and reduce unnecessary rescreening. Overall, the research underscores the importance of refining screening strategies for optimal neonatal hearing assessment.

### Factors Associated with Delayed TEOAEs Passing

GA and delivery methods emerged as significant factors affecting screening outcomes. Preterm neonates demonstrated a notable delay in passing TEOAE screening, with a 12.9-hour difference at the 90th percentile compared with term neonates. This delay likely reflects either cochlear functional immaturity or temporary fluid retention in the auditory pathway. Cesarean delivery was associated with increased passing ages at the 25th and 50th percentiles (22.3 and 28.8 hours, respectively) and modestly significant delay using Cox's regression analysis (HR: 0.77; 95% CI: 0.61–0.97; *p* = 0.024), though this effect diminished at higher percentiles. This pattern, consistent with large retrospective studies in China and Iran, 14 15 suggests that delayed amniotic fluid clearance or transient respiratory effects influence early screening outcomes in cesarean deliveries.

Regarding procedural factors, screening room 1 showed significantly better screening outcomes than other locations, likely reflecting institutional workflow rather than acoustic variations. Its proximity to the nursery ward makes it the typical location for initial screenings, while rooms near the maternal postpartum ward (rooms 2 and 3) more commonly conduct follow-up screenings. Monitored and corrected by the screening equipment, environmental noise levels showed no significant differences between locations, confirming that observed performance variations stemmed from screening timing and patient flow, not acoustic factors.

An intriguing discovery was the superior screening performance of SGA neonates at the 90th percentile compared with AGA neonates. This unexpected finding merits further investigation into potential physiological adaptations or methodological factors. Notably, several perinatal risk factors, including BW, APGAR scores (consistently ≥8), neonatal jaundice, and respiratory distress, showed no significant impact on screening outcomes, suggesting the robustness of current screening protocols.

These findings have important implications for clinical practice, particularly in establishing optimal screening protocols. The data support delaying screening beyond 48 hours to improve test reliability and reduce unnecessary re-screenings. However, the varying needs of preterm and cesarean-delivered neonates suggest the value of implementing personalized timing strategies for these populations.

### Strengths, Limitations, and Future Research

While this study benefits from a robust cohort size and comprehensive analysis of multiple factors, its single-center design presents inherent limitations. The generalizability of findings may be affected by specific population characteristics and institutional practices. Future research should focus on validating these findings through larger, multi-center studies to develop more refined and widely applicable screening guidelines, including cost-effectiveness analysis and a quality improvement protocol study.

## Conclusion

This study provides substantive evidence for optimizing NHS protocols through the strategic timing of TEOAE screening, particularly demonstrating superior outcomes when conducted after 48 hours of life. Our findings illuminate the importance of considering patient-specific factors, especially for preterm infants and those delivered via cesarean section, while revealing that considered perinatal risk factors had minimal impact on screening success. The research contributes valuable insights into screening optimization, including the unexpected superior performance of SGA neonates, suggesting opportunities for protocol refinement. While these results have immediate practical applications for healthcare institutions seeking to enhance their screening efficiency, the single-center nature of our study indicates the need for larger-scale, multi-center validation studies to develop more nuanced, population-specific screening guidelines. This research ultimately advances our understanding of optimal NHS practices, supporting the development of evidence-based protocols that can improve early hearing detection and intervention outcomes across diverse healthcare settings.
